# Update in Lung Cancer Molecular Pathology: Technological Advances and Clinical Practice

**DOI:** 10.3390/cancers15153812

**Published:** 2023-07-27

**Authors:** Paolo Graziano, Giulio Rossi

**Affiliations:** 1 Services Department, Pathology Unit, Scientific Institute for Research and Health Care (IRCCS) “Casa Sollievo della Sofferenza”, Viale Cappuccini 1, 71013 San Giovanni Rotondo, Italy; 2 Services Department, Pathology Unit, Fondazione Poliambulanza Hospital Institute, Via Bissolati 57, 25124 Brescia, Italy

This Special Issue of eleven articles, including six original works and five reviews, demonstrates the modern heterogenous approach to lung cancer by means of various methodologies from international experts from various countries. Lung cancer patients, particularly non-small-cell lung cancer (NSCLC), are indeed experiencing a dramatical change in the diagnostic and therapeutic phases, nowadays characterized by the advent of alternative and effective therapies based on the identification of molecular targets [1]. Among predictive biomarkers with deep impact in routine practice, An et al. [2] used selected reaction monitoring (SRM), a mass spectrometry-based proteomic method for quantitatively assessing predetermined candidate biomarkers in multiple samples, to detect ALK and other protein biomarkers NSCLC patients showing borderline or positive ALK FISH setup (Abbott ALK dual-color break-apart probe) but negative (22 cases among 4588 NSCLC) ALK immunohistochemistry (clones 5A4 and D5F3). SRM assay detected ALK protein in seven discordant cases (six out of eight fully positive and one out of thirteen borderline positive for ALK FISH), showing superior sensitivity in detecting the ALK protein in samples with poor fixation and or suboptimal preanalytical management. Of note, SRM has the possibility to quantitatively screen several protein targets, comprising ALK and MET, and FR-alpha, hENT1, RRM1, TUBB3, ERCC1, and XRCC1 for chemotherapy. Another original work by Leone et al. [3] described a multicentric experience using a novel, fully integrated and automated cartridge-based assay providing deparaffinization and digestion of the tissue up to mRNA amplification using real-time-PCR (Idylla, Biocartis) with the novel GeneFusion assay. The assay simultaneously identifies ALK, ROS1, RET and NTRK1/2/3 gene fusions and MET ex14 skipping mutations, and the results were matched with different techniques routinely available for predictive biomarker testing, either immunohistochemistry (IHC), fluorescence in situ hybridization (FISH), real-time-PCR (RT-PCR) or next-generation sequencing (NGS). Overall agreement was found in 92.3%, suggesting Idylla GeneFusion assay as a reliable tool to define druggable gene fusions and as a valuable stand-alone diagnostic test when time efficiency is needed or flanking NGS as an orthogonal method. In addition, tumor sections stored at room temperature for up to 60 days (17 cases were older than 2 years) were successfully characterized. Concerning EGFR mutations, Bironzo et al. [4] demonstrated the presence of co-mutations in 55% of 106 consecutive EGFR-mutated advanced NSCLC treated with first-line TKIs using a 22-gene-panel NGS. TP53 was the most common mutated gene (34%; 28 pathogenic), but several others were detected (CTNNB1 in 7.5%, PIK3CA in 5.7%, but also NRAS, MET, PTEN, AKT, SMAD4, RET, DDR2, FGFR3). Nevertheless, no significant differences in overall survival (22.8 vs. 29.5 months; *p* = 0.088), progression-free survival (10.9 vs. 11.2 months; *p* = 0.415) and overall response rate (55.9% vs. 68.1%; *p* = 0.202) were observed comparing patients with or without co-alterations. A greater rate of patients with EGFR exon 19 deletion harbored concomitant mutations (n:33; 70.2%) as compared with those showing EGFR exon 21 L858R mutation (n:10; 21.3%) without correlations to PD-L1 expression. Co-alterations were associated with younger age (*p* = 0.018) and baseline lymph nodes metastases (*p* = 0.032), whereas NSCLC patients without concomitant alterations had a significantly higher risk of bone progression (26.5% vs. 3.3%, *p* = 0.011). Of note, pathogenic EGFR co-mutations did not seem to predict survival nor efficacy of EGFR TKIs in previously EGFR-positive untreated advanced NSCLC. On the other hand, Verzè et al. [5] investigated the role of three different liquid biopsy platforms (plasma, urine, and exhaled breath condensate, EBC) as alternative sources of tissue to detect EGFR mutations in NSCLC patients. The authors enrolled 22 EGFR exon 20 T790M-mutated NSCLC patients in progression to first-line treatment and candidate to osimertinib. Plasma, urine and EBC samples were collected at baseline and every two months until progression and molecular analysis of cfDNA was performed using ddPCR and compared to tissue. A low sensitivity in urine and EBC samples, while the overall detection rate in the plasma showed a sensitivity of 58%. Although the detection of EGFR mutations in plasma anticipates radiologic progression, no improvement was evidenced when adding urine and EBC. Among oncogenic drivers in NSCLC, KRAS mutations represent the commonest genetic alterations (about 35%), particularly in smokers and the Caucasian population. In this Special Issue, the review by O’Sullivan et al. [6] highlighted the role of activated G12C-mutated KRAS isozyme that can be directly inhibited by a new class of selective small-molecule inhibitors, namely sotorasib and adagrasib, recently approved in locally advanced or metastatic KRAS G12C-mutated NSCLC patients who have received at least one prior systemic therapy. At least twelve KRAS G12C inhibitors are now available in clinical trials, either as a single agent or in combination and KRAS mutation prevalence is deeply analyzed. The authors also focused on the role of distinct mutations according to various histologies and clinical characteristics, and different mechanisms of KRAS inhibitors’ resistance limiting their use in combination treatment strategies. The advent of immunotherapy deeply impacted the therapeutic management of lung cancer patients, although a perfect predictive biomarker is still lacking. In the current Special Issue, several works from different perspectives have investigated this aspect. Augustus et al. [7] reviewed the role of liquid biopsy, referring to the tumor-derived material present in body fluids, offering an alternative approach to programmed death ligand 1 (PD-L1) level on tumor tissue and remembering how PD-L1 expression on tumor cells is the only diagnostic test approved by different regulatory drug agencies, although only about 20% of NSCLC patients significantly benefit from immunotherapy. Liquid biopsy is a less invasive technique providing real-time information on tumor characteristics. The authors focused on the role of circulating cell-free DNA (cfDNA), circulating tumor cells (CTCs), extracellular vesicles (EVs), epigenetic signatures, microRNA (miRNA), volatile organic compounds (VOCs) and the gut microbiota. The challenges and the opportunities of blood-based biomarkers, the T cell repertoire, and feces-based and breath-based biomarkers are addressed. Although promising, the authors underlined the need to validate this alternative approach within clinical trials, including larger cohorts, since the major hurdles consist of the low sensitivity and specificity of available detection techniques somehow related to the low abundance of most liquid biopsy compounds, especially in patients with localized tumors, the lack of standardized and harmonized protocols and the lack of reimbursement in many countries. Having said that, only a subset of NSCLC patients overexpressing PD-L1 (with tumor proportional score ≥50% at IHC) have a real benefit in first-line treatment from immunotherapy with pembrolizumab. Here, the monocentric study by Poma et al. [8] investigated the expression of 770 genes involved in the regulation of the immune system using nanoString technology. Among 46 consecutive advanced NSCLC patients (negative for EGFR, ALK, ROS1, BRAF, MET and ERBB2 genes, with PD-L1 higher than 50% of tumor cells) treated with pembrolizumab in a first-line setting, durable clinical response was observed in tumors, showing greater cytotoxic cell infiltration and exhausted CD8, B-cells, CD45, T-cells, CD8 T-cells, and NK cells. Immune cell scores such as CD8 T-cell and NK cell were good predictors of durable response (area under the curve, AUC = 0.82), more consistent than PD-L1 overexpression (AUC = 0.61). Among the immune cell markers, XCL1/2 showed better performance in predicting durable benefit to pembrolizumab (AUC = 0.85). Of note, CD8A/B and EOMES showed a high specificity (>0.86) in identifying responders to pembrolizumab. Finally, the authors demonstrated that the use of single CD8 T-cell and NK cell markers such as CD8A/B and XCL1/2 could represent a simple and effective strategy for clinical practice. At the same time, Granata et al. [9] analyzed the role of the radiomic approach obtained via computed tomography (CT) examination in assessing the efficacy of immunotherapy in lung adenocarcinoma. A total of 573 radiomic metrics were extracted, and the inclusion criteria were satisfied for 38 patients subjected to immunotherapy. The shift in the center of mass of the lesion due to image intensity was significant both in predicting overall and progression-free survival in patients subjected to immunotherapy and in patients of the control group. Regarding multivariate analysis, considering the robust (two morphological features, three textural features and three higher-order statistical metrics) application of the least absolute shrinkage and selection operator (LASSO) method and all patients, a support vector machine reached the best results for stratifying patients based on OS (AUC = 0.89; accuracy: 81.6%), then demonstrating that specific radiomic features extracted by CT could predict immunotherapy efficacy in lung adenocarcinoma. Since up to 40% of patients with NSCLC experienced brain metastasis in the course of the disease, Souza et al. [10] reviewed the mechanisms underlining the metastatic spread of lung cancer to the brain. Brain metastasis seems to arise via the seeding of circulating tumor cells into the brain microvasculature. The interaction of tumor cells with immune cells might promote a microenvironment favorable to cancer cell growth. The authors highlighted the molecular factors promoting brain metastasis in terms of genomic and transcriptomic changes, including coding and non-coding RNAs, finally overviewing the current and novel therapeutic strategies available for NSCLC patients diagnosed with BM. The last articles of this Special Issue concern the promising role of extracellular vesicles (EVs) in lung cancer. These nano-sized lipid-bound particles ranging from exosomes (30–150 nm), microvesicles (100–1000 µm) to apoptotic bodies (1–5 µm), contain proteins, nucleic acids, and metabolites. EVs are also released by tumor cells during different phases of tumor progression from neoplastic development to drug resistance and may be detected in several fluids, including blood, saliva, sputum and effusions in various tumor stages. Kato et al. [11] underlined the role of EVs derived from cancer and immune cells mediating immunomodulatory functions. The review analyzed the biogenesis, components, biological functions and isolation methods of EVs and their potential clinical utility for diagnostic and therapeutic applications in lung cancer. Similarly, the review article by Sandua et al. [12] focused on the role of exosomes as microvesicles actively secreted by tumor cells and involved in lung cancer development. Exosomes include several molecules, from proteins to RNA and DNA, creating a background for promoting lung carcinogenesis and favoring intercellular signals for tumor growth, metastasis, epithelial-to-mesenchymal transition, neoangiogenesis, immunosuppression and even drug resistance. Since liquid biopsy using plasma is commonly used for the identification of druggable genetic alterations, this could be the most available material to investigate the role of exosomes in lung cancer. Detection technologies for exosome isolation and their analysis are also discussed.

This series of articles basically represents the heterogeneous approach to diagnosis and treatment from different specialties that nowadays characterize the scientific community involved in lung cancer research. Some data here are currently applied in routine practice, while others represent a promising future scenario, mainly based on investigations of cancer-related molecules present in several liquid biopsies. We hope that at least some of the evidence provided by the authors of this Special Issue may be of some interest to readers, possibly switching on new ideas for improving the management of lung cancer patients.
